# Mitral Annular Disjunction: Pathophysiology, Pro-Arrhythmic Profile and Repair Pearls

**DOI:** 10.31083/j.rcm2304117

**Published:** 2022-03-30

**Authors:** Dimos Karangelis, Konstantinos S. Mylonas, Argyris Krommydas, Spiros Loggos, Vasiliki Androutsopoulou, Dimitrios Stakos, Dimitrios Mikroulis, Aphrodite Tzifa, Fotios Mitropoulos

**Affiliations:** ^1^Department of Cardiac Surgery, Democritus University of Thrace, University Hospital of Alexandroupolis, 68100 Alexandroupoli, Greece; ^2^Third Department of Cardiac Surgery, Onassis Cardiac Surgery Center, 10671 Athens, Greece; ^3^Department of Echocardiography, Mitera Hospital, 15123 Athens, Greece; ^4^Department of Cardiac Surgery, Mitera Hospital, 15123 Marousi, Athens, Greece; ^5^Cardiology Department, University Hospital of Alexandroupolis, Democritus University of Thrace, 68100 Alexandroupolis, Greece; ^6^Department of Congenital Cardiology, Mitera Hospital, 15123 Marousi, Athens, Greece; ^7^School of Biomedical Engineering & Imaging Sciences, King’s College London, NW3 3 London, UK

**Keywords:** mitral disjunction, mitral valve prolapse, mitral regurgitation, lethal arrhythmias, ventricular arrhythmias, mitral valve repair, mitral surgery

## Abstract

Mitral annular disjunction (MAD) is a structural abnormality defined by a distinct separation of the mitral valve annulus—left atrial wall continuum and the basal aspect of the posterolateral left ventricle. This anomaly is often observed in patients with myxomatous mitral valve prolapse. Importantly, MAD has been strongly associated with serious ventricular arrhythmias and predisposes to sudden cardiac death. Therefore, we have to emphasize the need to diagnose this morphologic and functional abnormality in routine practice in order to facilitate optimal mitral valve repair and minimize patient risks. Nevertheless, clinical knowledge regarding MAD still remains limited. In the present review, we aim to shed light on several aspects of MAD, including distinct anatomical and pathophysiological characteristics, imaging modalities, association with ventricular arrhythmias, and current methods of treatment.

## 1. Definition and Anatomical Considerations

Mitral valve prolapse (MVP) is the most frequent cause of primary mitral regurgitation (MR) affecting 2.4% of the general population [1]. The observed leaflet redundancy which is regarded as the main structural abnormality present in MVP, was thought to be the only mitral valve-related aberration that could predispose to complex ventricular arrhythmias and sudden cardiac death [2]. Recently, another type of mitral pathology termed mitral annular disjunction (MAD) was reported in patients with MVP and complex arrhythmias [3]. MAD is a cardiac structural abnormality characterized by a distinct separation of the mitral valve annulus—left atrial wall apparatus and the basal aspect of the posterolateral left ventricle (LV) [4]. Disjunction was first described in 1981 by Bharati *et al*. [5] in a brief communication paper delineating the case of a 45-year-old patient with a floppy mitral valve who died suddenly after a long history of palpitations [5]. Five years later, Hutchins *et al*. [6] identified MAD in 92% of 25 heart autopsies with mitral valve prolapse (MVP). At that time, MAD was thought to be of little clinical consequence and received little attention [6]. In the 90s MAD became a theoretical and speculative matter in pathological reports [7]. Subsequently, it started to gain interest due to the fact that routine transthoracic echocardiography made it easy to detect and quantify MAD. One important preliminary paper which introduced echocardiography in the assesment of MAD was the report by Carmo and colleagues [4]. The authors found that the function of the mitral annulus was substantially impaired in patients with MAD and also correlated the severity of MAD with the occurrence of non-sustained ventricular tachycardia [4].

To this day, the pathophysiology of MAD is still not fully understood. It is well known, however, that the mitral valve (MV) annulus is not actually a discrete, fibrous, ring-like structure but rather represents the attachment line of mitral leaflets to the atrioventricular junction. In this context, the mobility and pattern of contraction of the MV annulus is dictated by the LV contractility and the aortic root [8]. Therefore, in regular circumstances, the MV annulus moves in systole towards the LV apex and in diastole towards the left atrium [9].

In the presence of MAD, the annulus is functionally disengaged from the left ventricle, and a paradoxical annular movement occurs as the annulus moves consistently with the left atrium during the cardiac cycle (instead of the LV). Expanding and flattening of the annulus occurs in systole, causing the segment of the left ventricular wall which is adjacent to the disjunction area to move outwards in systole and inwards in diastole. This prominent flattening of the MV annulus in systole imposes mechanical stress on the leaflets of the valve as well as the chordae tendineae, which can lead to valvular degeneration [10].

The aorto-mitral continuity (aorta and the anterior MV leaflet) is less prone to dilation due to the support of two robust fibrous trigones. On the other hand, the posterior part of the MV annulus appears to be significantly more susceptible to the effects of mechanical stress. These features largely explain why MAD affects the territory directly under the posterior MV leaflet (specifically the P1 and P2 scallops) [11, 12]. MAD has a dynamic nature and it is detectable in systole as the myocardium of the ventricle contracts. This nature of the ventriculoannular detachment explains the paucity of pathological studies on flaccid hearts [6]. It is also quite obvious why surgeons may not notice this anatomical variation unless the posterior leaflet is separated.

## 2. Incidence and Pathophysiology

MAD accompanies various types of mitral pathology. In a 2019 systematic review that included 19 studies, the pooled incidence of MAD was estimated to be 51% in patients with myxomatous mitral valves, 32.6% in the context of MVP, and 25.9% in severe mitral valve regurgitation and floppy MV [13]. Severe myxomatous disease involving bileaflet MVP and marked leaflet redundancy have been independently associated with annular disjunction [14].

The pathophysiology behind disjunction and the reason why it varies in incidence among different patient groups has still to be defined. To date, it remains unclear whether MAD constitutes an acquired structural abnormality or it has a congenital substrate. As delineated above, there is a higher proportion of patients with MAD who have a myxomatous mitral valve compared to patients with a structurally normal heart [13]. Some speculate that substantial mechanical stress and stretch placed upon the MV annulus and apparatus favor excess tissue formation and leaflet mobility, ultimately resulting in billowing and prolapse [6].

## 3. Diagnosis and Imaging

It should be emphasized that the diagnostic cut-off for disjunction is not unanimously accepted. In the original histological report by Hutchins *et al*. [6] wide separation (>5 mm) was required to diagnose MAD. This description was initially adopted in two-dimensional (2D) [12, 15] and three-dimensional (3D) *transesophageal* echocardiographic (TEE) studies [10]. However, in recent years a threshold of >2 mm for two-dimensional transthoracic echocardiography (TTE) measurements was proposed and is gradually gaining traction [16].

Taking it a step further, Tani *et al*. [17] classified disjunction according to the degree of separation, as follows: type 0 in which no MAD is apparent, type I which refers to a hypermobile basal left ventricular segment and no MAD, type II which corresponds to MAD less than 5 mm, and type III in which MAD is more than 5 mm [17]. MAD can be diagnosed using non-invasive imaging, including TTE or TEE studies, computed tomography (CT), and cardiac magnetic resonance (CMR). By definition, MAD is seen only in systole when the posterolateral portion of the LV contracts and the MV annulus “slides” thereby detaching from the LV myocardium.

When transthoracic echocardiography is utilized, MAD is assessed by measuring the distance from the site of the posterior leaflet insertion into the left atrial wall, which corresponds to the upper border of the disjunction, to the point where the left atrium associates with the ventricular myocardium (lower border of the disjunction). This is best achieved in a parasternal long axis TTE view at end-systole. By means of 2D TTE, the degree of annular displacement can be best measured at the P2 level by using a 4-chamber mid-esophageal view at 0 degrees during systole [12].

CMR not only has high sensitivity in identifying MAD but can also provide instrumental data regarding the distribution and extent of myocardial and papillary muscle fibrosis [18]. Interestingly, Dejgaard *et al*. [19] utilized CMR and found that the circumferential extension of MAD ranges between 30^∘^–240^∘^ (median 150^∘^), meaning that MAD can take up to 2/3 of the annular circumference [19]. Lastly, cardiac CT has also been used to confirm the presence and quantify the degree of MAD by rotating the view plane around the center of the MV to visualize the disjunction along the annular circumference [20].

## 4. The Impact of MAD on MVP

According to some authors MAD has been considered to precede occurrence of MVP [6], while others support that it is developed either independently of MVP [16, 19] or even as a side-product of myxomatous MVP [12]. MAD prevalence and associated MVP phenotypes were recently analyzed in a large cohort of 595 patients with isolated MVP [14]. Besides the common presence of MAD in patients with MVP (31%), the authors reported that advanced myxomatous degeneration characterized by marked leaflet redundancy and bileaflet prolapse was the most dominant feature of MVP in MAD [14]. The frequency of bileaflet prolapse in patients with disjunction has also been described by Mantegazza and colleagues, who further noted that patients with MVP have been shown to develop significant mitral regurgitation at an earlier age when MAD is present [11]. Furthermore, the researchers noted that the incidence of MAD was higher in patients with Barlow’s disease compared to fibroelastic deficiency (22% vs 6%), although the maximum distance of MAD was similar between these two phenotypes of degenerative mitral disease [11].

A 2021 study by the Mayo Clinic assessed annular, valvular and ventricular dynamics in MVP with severe regurgitation stratified by presence of MAD [21]. Patients with evident MAD had significantly larger diastolic annular areas (mean, 1646 ± 410 vs 1380 ± 348 ${\mathrm{mm}}^{2}$), circumferences (mean, 150 ± 19 vs 137 ± 16 mm), and intercommissural diameters (mean, 48 ± 7 vs 43 ± 6 mm) compared to those without disjunction. Moreover, the mid- and late systolic excess intercommissural diameter, circumference enlargement, and annular area were significantly linked with MAD. Additionally, MAD was associated with dynamically annular slippage, a statistically significant larger prolapse volume and height (*p *≤ 0.007), as well as a larger leaflet area (mean, 2053 ± 620 vs 1692 ± 488 ${\mathrm{mm}}^{2}$, *p* = 0.01) [21].

Moreover, it has been shown that the incidence of chordal rupture was reduced in patients with MAD and MVP (52–61%) compared to prolapse alone (73–75%) [10, 11]. Despite the fact that the presence of MAD did not affect ejection fractions/LV strain, in the setting of disjunction, the systolic basal posterior thickness was observed to be increased (mean, 19 ± 2 vs 15 ± 2 mm, *p *< 0.001), with higher systolic thickening of the basal posterior wall (mean, 74 ± 27% vs 50 ± 28%) and greater ratio of basal wall thickness to diameter (*p *≤ 0.01) [11].

## 5. Association with Ventricular Arrhythmias

A growing body of literature has shown a strong association between ventricular arrhythmias and MAD. Impressively, 15% of patients with cardiac arrest and no identifiable cause seem to have underlying MAD (which may have precipitated the event) [13]. Furthermore, patients with more extensive MAD and circumferential area seem to carry an even greater risk of ventricular arrhythmias [19, 22]. Indeed, a disjunction of >8.5 mm has been shown to strongly predispose to ventricular tachycardia (OR: 10; 95% CI: 1.2–78.1) [4].

Late gadolinium enhancement suggests myocardial fibrosis and scarring, which may further predispose to ventricular arrhythmias. A study by Perazzolo Marra and colleagues reported that there was a higher extend of late gadolinium enhancement within the LV with greater MAD diameters in patients who suffered sudden cardiac death [23]. Essayagh *et al*. [22] also associated MAD with ventricular arrhythmias and reported that late gadolinium enhancement was detected within the papillary muscles in 84% of the patients [22]. In a more contemporary report, the same group also suggested that MAD over time contributes significantly and independently to arrhythmic MVP occurrence likely due to progressive fibrosis of the mitral apparatus [24].

Dejgaard *et al*. [19] conveyed that gadolinium enhancement in papillary muscles (OR: 4.09; 95% CI: 1.28–13.05), as well as increased longitudinal MAD distance in the posterolateral LV wall (OR: 1.16; 95% CI: 1.02–1.33) are predictive of ventricular arrhythmias. In their series, late gadolinium enhancement in the anterolateral papillary muscle was firmly combined with severe arrhythmic events (OR: 7.35; 95% CI: 1.15–47.02) [19]. Syed and coauthors also suggested that excessive mobility of the basal anterolateral and posterolateral LV segments may generate a greater degree of mechanical stress on mitral valve annulus and therefore produce myocyte hypertrophy and fibrosis. Subsequently, this may induce electrical instability [25]. Picklehaube’s sign refers to lateral MV annular systolic velocity more than 16 cm and is observed with excessive pulling of the posterolateral LV wall. This sign may be valuable in detecting substantial hypermobility which may predispose patients with MAD to ventricular arrhythmias [26].

Although MAD appears to be profoundly pro-arrhythmic, the diagnosis of isolated disjunction should not affect clinicians to acknowledge this as an imminent risk factor for sudden death in all patients. Essayagh *et al*. [24] in their latest study confirmed that survival after MVP diagnosis was non-inferior in patients with MAD in the first 10 years following diagnosis. Although this may seem reassuring, careful follow up is warranted in most cases.

## 6. Surgical Repair in Mitral Regurgitation with MAD

**Fig. 1.**
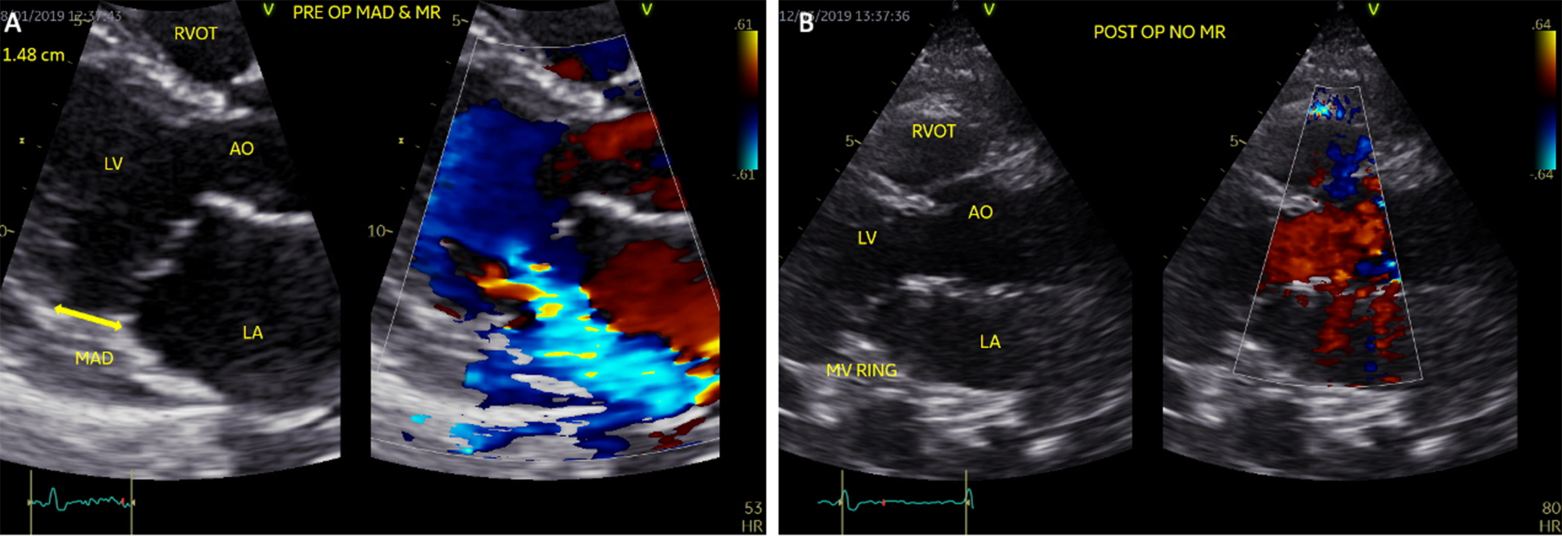
**Transthoracic echocardiogram of a 60-year-old female patient**. (A) Preoperative views showing mitral annular disjunction (left) and severe mitral regurgitation and during systole (right). The distance between mitral valve leaflet-atrial wall and left ventricle is measured 1.48 cm (yellow bidirectional arrow). (B) Postoperative views after mitral valve repair with a 34 mm ring. No mitral annular disjunction is identified. No mitral regurgitation is detected. LV, Left Ventricle; LA, Left Atrium; AO, Ascending Aorta; RVOT, Right Ventricular Outflow Tract.

MAD should be thoroughly investigated during assessment of severe MR in MVP as its recognition is important to achieve optimal surgical repair. Careful surgical planning and modification of the repair technique, once the surgeon is aware of MAD is imperative in these challenging cases. Mitral valve repair can establish complete postoperative MAD resolution. This is achieved by suturing a ring which affixes the annulus to the ventricular myocardium and collapses the MAD area (Fig. 1).

The key determinant of a successful mitral repair in these cases is to firmly suture the ring to the ventricular myocardium in the area of the pre-operative MAD. It has been shown that MAD per se, does not hinder the feasibility and quality of valve repair [21]. Nevertheless, some patients may be left with a degree of residual MAD after the repair due to incomplete ring sutures that do not properly affix the annulus to the posterior wall but rather attach it to atrial wall. In these cases, it is not yet fully clarified whether persistence of MAD post-repair may lead to progressive LV fibrosis and arrhythmia [21]. Having said that, in the study by Essayagh *et al*. [14] successful mitral surgery was associated with a trend towards lower rates of observed arrhythmias. More specifically, the authors report that the link between MAD and arrhythmic events was strong under medical management (adjusted HR: 3.21; 95% CI: <2.03–5.06; *p *< 0.0001) but was weaker after mitral surgery (adjusted HR: 2.07; 95% CI: 1.24–3.43; *p* = 0.005) [14].

Historically, Tirone David’s group first proposed that to make mitral repair successful in the setting of MAD, the posterior leaflet has to be detached and reattached to the proximal musculature of the LV and then secured with an annuloplasty ring [15]. Based on the specific pathology at hand, either the entire posterior leaflet or just P2–P3 are detached from their insertion. Moreover, the same group proposes a liberal use of artificial chordae as a means to increase the durability of the repair.

Although David’s group favors flexible bands for stabilization of the posterior annulus [15], Carpentier has used exclusively rigid rings to successfully reattach the posterior leaflet to the endocardium of the LV in patients with calcification of the annulus and MAD [27]. Using the Toronto repair approach, freedom from valve-related morbidity and mortality at 1, 5, and 10 years was 94% ± 2%, 90% ± 2%, and 78% ± 4%, respectively, while the event-free survival was 94% ± 1.6%, 89% ± 3%, and 75% ± 5%. Based on these findings, mitral valve repair in the setting of MR with advanced myxomatous degeneration and valvular disjunction seems to be enduring but not as enduring as for isolated prolapse of the posterior leaflet [28]. Interestingly, it seems to mirror more the outcomes of repair for bileaflet or anterior leaflet prolapse. With regards to rate of reoperation, this was low, considering that hardly a 3% of the patient population presented severe recurrent MR. Nevertheless, 11.6% of the patients exhibited moderate MR at follow-up, indicating that MV repair may decelerate but does not halt the degenerative process altogether.

Mayo Clinic data also showed that MAD receded following mitral valve repair, and parameters such as LV diameter and wall thickening had no difference between patients with and without MAD [21].

With regards to which is the most beneficial and effective method of surgery (i.e., repair or replacement) in terms of postoperative arrhythmic events there is still not enough data to support either approach. Both surgical procedures however, can achieve complete disappearance of MAD in the postoperative setting in almost all patients, and have demonstrated to reduce the burden of malignant arrhythmias in MVP patients. This is probably because either the ring or the prosthesis (in the case of replacement), when sutured, will join the annulus to the LV myocardium, and collapse the area of disjunction [12, 14].

## 7. MitraClip in Mitral Regurgitation with MAD 

MitraClip may represent a reasonable palliative method. Indeed, patients with severe regurgitation, extensive myxomatous degeneration, low ejection fraction, and myocardial fibrosis may be poor surgical candidates and could benefit from percutaneous edge-to-edge repair via MitraClip implantation. Of note, the new version of MitraClip (XTR) has increased arm length (12 mm from 9 mm), which improves coaptation and facilitates grasping by two additional sets of frictional elements at the grippers. Due to its technical characteristics, it can be applicable in cases of prolapse with redundant leaflet tissue, and it has already been reported to be favorable in advanced myxomatous mitral disease with MAD [29, 30].

There is a relative dearth of literature regarding the effect of mitral valve repair with Mitraclip on the occurrence of ventricular arrhythmias. Use of MitraClips, as shown in the prospective study by Ledwoch *et al*. [31] has led to a substantial decrease of ventricular arrhythmias in a cohort of 50 heart failure patients with severe MR. MitraClip implantation clearly adds to the reduction of MR, and improvement of LV function [30]. This reduction alone however, achieved by the edge-to-edge repair, may not be sufficient in the long run in the presence of MAD. The annular correction during surgical repair does not occur with transcatheter edge-to-edge mitral repair. It seems rather reasonable that patients with severe MR and MAD will benefit more from surgical repair, however this has yet to be established. Additional future studies are warranted to clarify whether additive annular therapy for MAD is necessary in cases dealt with Mitraclips.

## 8. Conclusions

MAD is a structural aberration defined by a specific disengagement of the mitral valve annulus—left atrial wall continuity and the basal aspect of the posterolateral LV. It is frequently encountered in patients with myxomatous mitral valve degeneration and MVP. On imaging, MAD is only recognizable during ventricular systole. Although initially thought to be of little clinical importance, a growing body of literature has associated the presence of disjunction with ventricular arrhythmias and sudden cardiac death. Surgical MV repair is the standard of care for patients with severe regurgitation. To ensure a durable repair in the setting of MAD, the posterior leaflet has to be detached and reattached to the proximal musculature of the LV and then secured with an annuloplasty ring. Patients with low contractility reserves and myocardial fibrosis may be considered for palliative percutaneous edge-to-edge repair using MitraClip technology. Irrespective of the repair approach, it is still unclear whether and to what extent long-term outcomes are affected by annular disjunction.
